# Treating to Target in Clinical Practice: The Results of a Questionnaire Completed by Greek Rheumatologists

**DOI:** 10.31138/mjr.31.1.145

**Published:** 2020-05-25

**Authors:** Theofilos-Diamantis Karatsourakis, Xenofon Baraliakos

**Affiliations:** 1 AbbVie Pharmaceuticals S.A., Athens, Greece; 2 Rheumazentrum Ruhrgebiet, Ruhr-University Bochum, Herne, Germany

**Keywords:** rheumatoid arthritis, spondyloarthritides, treat to target

## Abstract

**Background:**

The Treat-To-Target (TTT) approach is an important part of clinical practice in rheumatology. Although this approach is well structured in rheumatoid arthritis (RA) and data are accumulating in psoriatic arthritis (PsA) and axial spondyloarthritides (AxSpA), the systematic application of this process in clinical practice can be further improved to achieve better treatment outcomes.

**Objective:**

The aim of this study was to present the perspective of clinical rheumatologists on how they evaluate disease activity, in what patient groups they regularly use treatment targets, and how they prioritize treatment targets in spondyloarthritides.

**Methods:**

A questionnaire consisting of eight questions on the management of RA, PsA and AxSpA (4 focusing on the use of indexes when setting treatment targets, 2 on the frequency and the patient groups to which these are being applied, and 2 on the physician’s priorities in managing different manifestations of the SpA spectrum) was completed by private practice and hospital-based rheumatologists.

**Results:**

160 rheumatologists completed the questionnaire. The majority use the formal composite indexes in clinical practice, certain items from these indexes, and patients’ evaluation of the treatment intervention. Indexes are applied frequently in most patient groups, and the priorities of rheumatologists include both musculoskeletal manifestations, as well as the other clinical aspects of the SpA spectrum.

**Conclusion:**

The results can contribute to the understanding of the adherence of rheumatologists to this TTT strategy.

## INTRODUCTION

Rheumatoid arthritis (RA), psoriatic arthritis (PsA) and axial spondyloarthritides (AxSpA) are chronic autoimmune diseases of unknown aetiology with no available cure.^1–3^ The current treatment options aim to abrogate inflammation as clinically manifested in the affected tissues and organs, and control the structural damage through a treat-to-target approach.^4–5^ This strategy is gradually being implemented in RA, after proven benefits have been shown in diseases like diabetes mellitus and arterial hypertension, aiming at an optimisation of outcomes in patients with chronic diseases with increasing burden in the course of the disease. The treat-to-target strategy is thoroughly documented in RA by an extensive bibliography, including the long-term benefits from its application^6^; however, in PsA and AxSpA, the same principles from this strategy are extrapolated, and more data are expected to refine it and document the benefits.^5^ The incorporation of various disease indexes in clinical practice, including their limitations, is well established in RA.^7^ On the other hand, an active discussion of experts is ongoing in both PsA and AxSpA - multifaceted diseases with different manifestations - including the natural course in each individual patient.^2,8^ Of note, the recent guidelines and recommendations of the American College of Rheumatology, in collaboration with the National Psoriasis Foundation for the Treatment of Psoriatic Arthritis and the Spondylitis Association of America/Spondyloarthritis Research and Treatment Network for the Treatment of Ankylosing Spondylitis and Nonradiographic Axial Spondyloarthritis, indicate a different approach.^9,10^ The conditional recommendation for PsA is to use a treat-to-target strategy without a clear proposal for a specific index-based target, indicating that a patient-doctor discussion may define it individually. However, in AxSpA, the recommendation is against using an Ankylosing Spondylitis Disease Activity Score (ASDAS)-based treat-to-target strategy in favour of a physician-based assessment, and the expert panel judged that more convincing evidence is required to stablish the benefits and risks of treating to target in the context of fewer therapeutic options.

Considering the complexity of management of any chronic illness, important changes in management have shaped the care of patients with these chronic rheumatic diseases. In the last two decades, early diagnosis and new treatment options, combined with the adoption of the treat-to-target approach, have resulted in the current improved level of care, including in Greece. Inclusion of these scientific data in the therapeutic protocols of the Greek Rheumatology Society and Professional Association of Rheumatologists in each disease that are widely used in clinical practice, including the e-prescription platform in recent years, also have contributed to this improved care.^11–13^ The Greek scientific community has created databases with publications that describe the real-world treatment of patients within this framework,^14–18^ including the achievement of different levels of disease activity in patients with RA.^16^

A thorough understanding of the application of therapeutic protocols in clinical practice can potentially contribute to further improving the outcomes as agreed upon by doctors and patients. Part of the challenge in the long-term management of RA, PsA and AxSpA is that individualised therapeutic choices of rheumatologists and the needs and preferences of patients must be continuously adapted and combined in a complex setting of a healthcare services.

In RA, various indexes to describe the disease status have been defined for clinical trials and clinical practice by international scientific organisations. The trend towards using indexes that are more stringent and simple to use in various settings reflects the ambition to further elevate the level of standards of care, especially as more therapeutic options become available. In this manuscript, we describe the current priorities, the way each disease status is being evaluated, and the extent of application of treat-to-target strategy as described by the perception of clinical rheumatologists.

## MATERIALS AND METHODS

### Design

The questionnaire consisted of eight questions that had been created with by the authors along with community rheumatologists. There were six questions on the management of RA, PsA and AxSpA, including the principles of management of these chronic diseases, the parameters used to evaluate disease activity in each disease, the clinical context of using disease activity indexes and the extent of use of the treat-to-target strategy in clinical practice. Each question had six possible answers, and each participant in the survey could indicate only the one most appropriate. Two additional questions focused on the clinical priorities of various articular and extra-articular manifestations of PsA and AxSpA when managing patients, and each participant could choose up to two of the six possible answers. The questionnaire was completed digitally after each participant provided consent for the collection of answers and public presentation of the aggregated results. To analyse the answers to this questionnaire, each participant was first classified based either on the setting of workplace or on the area of residency.

### Statistical analysis

The answers to the questions were collected in the digital interface in a Microsoft Excel file template. They then were anonymised, aggregated, and subsequently analysed using Microsoft Excel functionalities. Only descriptive results were reported for the answers to each question, either in the total sample or in subgroups based on workplace or residency. No comparisons or statistical correlations were tested.

## RESULTS

**Figure 1.**
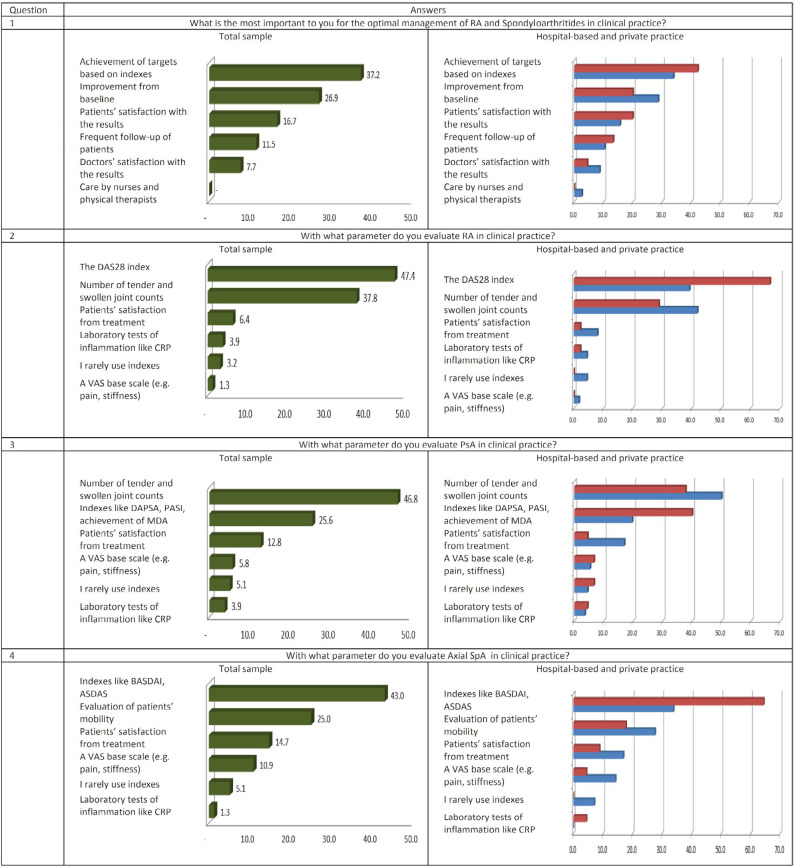

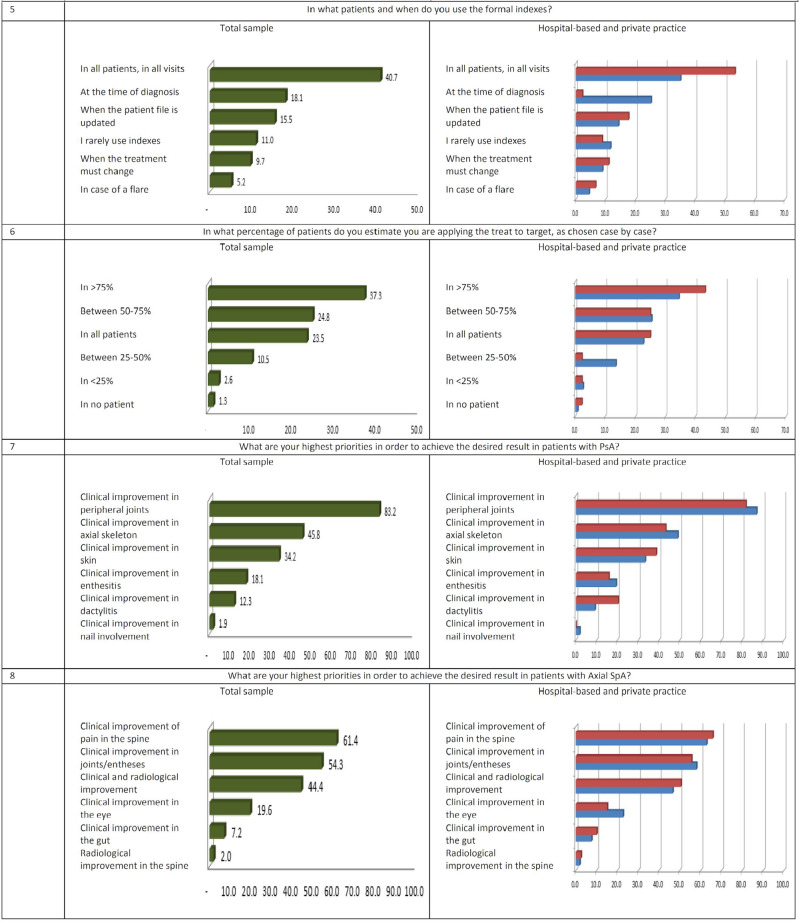


A total of 160 rheumatologists gave consent and completed the questionnaire. Of these, 72% were in private practice and 28% were hospital-based; 48% resided in urban areas and 52% resided in rural areas. In urban areas, the percentages of private practice and hospital-based rheumatologists were 64% and 36%, respectively; in rural areas, these percentages were 80% and 20%, respectively. The questions and responses follow (see *Figure 1* for detailed results).

### 

#### Question 1: What is the most important to you for the optimal management of RA and Spondyloarthritides in clinical practice?

Among the total sample of rheumatologists and in order of preference, achievement of an index-based targets, improvement from baseline and patient satisfaction were ranked highest, while patient frequent follow-up and physician satisfaction were ranked lower. Compared with rheumatologists in private practice, more hospital-based rheumatologists valued the achievement of index-based targets and patient satisfaction and fewer valued the perceived improvement from baseline. Differences based on residence were small.

#### Question 2: With what parameter do you evaluate RA in clinical practice?

Among the total sample, nearly half chose the Disease Activity Score in 28 joints (DAS28) index, while more than one-third chose evaluation of tender and swollen joint counts. Most hospital-based rheumatologists chose the DAS28 index, while most private practice rheumatologists chose tender and swollen joint counts or the DAS28 index, in that order.

#### Question 3: With what parameter do you evaluate PsA in clinical practice?

Both in the total sample, as well as by place of work and residence, most rheumatologists chose tender and swollen joint counts, followed by formal indexes of disease activity. Rheumatologists in private practice considered patient satisfaction more frequently than those hospital-based. However, among the total sample, patient satisfaction was chosen more frequently in PsA compared with RA.

#### Question 4: With what parameter do you evaluate axial SpA in clinical practice?

Among the total sample, rheumatologist preferences were more balanced: 42.9% chose indexes, 25% chose evaluation of patient mobility, 14.7% chose patient satisfaction and 10.9% chose Visual Analogue Score (VAS) for evaluation of pain and stiffness. Most hospital-based rheumatologists chose formal indexes, while those in private practice chose all of the above in a more balanced pattern.

#### Question 5: In what patients and when do you use the formal indexes?

Among the total sample, most rheumatologists chose formal indexes in all patients at every visit (40.7%), followed by time of diagnosis (18.1%) and when they update patient files (15.5%). Eleven percent answered that they rarely use indexes in their clinical practice. More than half of hospital-based rheumatologists chose indexes at every visit, when they update patient files and when they change treatment, while responses of those in private practice were more balanced; most notably, approximately one-fourth chose indexes at the confirmation of diagnosis. Nearly half of rheumatologists in urban areas chose indexes at every visit, while one-third in rural areas chose indexes at diagnosis.

#### Question 6: In what percentage of patients do you estimate you are applying the treat to target, as chosen case by case?

Among the total sample, most rheumatologists (37.3%) responded that they treat to a specific target in >75% of patients, with this percentage appearing to be numerically slightly higher in hospital-based versus private practice, and another 24.8% in 50–75% of patients.

#### Question 7: What are your highest priorities in order to achieve the desired result in patients with PsA?

For this question, up to two answers were accepted. The areas of priority clinical improvement among the total sample are as follows: peripheral joints (83.2%), axial involvement (45.8%) and skin manifestations (34.2%). The differences in subanalysis by groups are small.

#### Question 8: What are your highest priorities in order to achieve the desired result in patients with AxSpA?

For this question, up to two answers were accepted. The areas of priority clinical improvement among the total sample are as follows: pain in the spine (61.4%), peripheral involvement (joints/entheses; 54.3%) and achievement of clinical and radiological outcomes (44.4%). The differences in subanalysis by groups do not seem to be large for this question either.

## DISCUSSION

After years of implementation of the Greek Rheumatology Society’s therapeutic protocols for RA, PsA and AxSpA in clinical practice, and their inclusion in the e-prescription in the last years, a large percentage of rheumatologists are now recording the way they manage patients in everyday clinical practice in this framework. As more real-world data describing patients with RA, PsA and AxSpA are being published in Greece, both from individual academic centres and nation-wide databases, the areas where the management of patients with chronic rheumatic diseases can be further improved are becoming apparent.

### What are the tools?

Based on this questionnaire, the most important aspect of long-term management of chronic rheumatic diseases by rheumatologists is the achievement of index-based targets (37.2%), followed by improvement from baseline (26.9%) and patient satisfaction (16.7%).

The tools used to evaluate disease activity in clinical practice include formal indexes, such as DAS28 in RA and Bath Ankylosing Spondylitis Activity Disease Activity Index/Ankylosing Spondylitis Disease Activity Score (BASDAI/ASDAS) in AxSpA; the monitoring of main clinical manifestations, such as tender and swollen counts, mostly in PsA and secondarily in RA; and the evaluation of spine mobility in AxSpA. Based on questionnaire responses, patient satisfaction and VAS-based reporting of pain and stiffness, aspects that are more important in patients with AxSpA, are prioritised by as many as one-fourth of rheumatologists.

The discussion on the most appropriate tools and indexes to use in research is ongoing. The joint American College of Rheumatology/European League Against Rheumatism remission criteria in rheumatoid arthritis for clinical trials^19^ are not completely implemented in all settings, and clinical practice poses additional challenges. Nevertheless, based on the establishment of assessment of tender and swollen joint counts as part of routine visits in most patients, the addition of less time-consuming questions on the global assessments of the disease by patients and physicians requires a small additional effort to the thorough evaluation of patients with RA in clinical practice. This small added effort might lead to a qualitative stepping up in the implementation of formal tools.

### How does one use the tools?

Among the total sample of rheumatologists, 40.7% chose indexes of disease activity at every visit; the rest chose indexes when they update patient files, considered changing the current treatment or in other instances of less frequency. These results, when correlated with the previous answers, indicate that about 40% of rheumatologists accept the concept of the method and tools of treating with a target systematically and use them in clinical practice. When rheumatologists are asked how often they actually apply a strategy, with the chosen target used case by case, about 6 out of 10 report applying this in >75% of their patients. This difference may imply that using other targets or parameters than the formal indexes of disease activity, along with a structured process of disease monitoring.

Rheumatologists in hospital-based settings chose formal indexes more frequently and systematically both in RA and SpA, while those in private practice considered the number of tender and swollen joint counts and patient satisfaction more frequently, which may indicate that formal and more complex tools are easier to implement in tertiary care while simpler and patient-centred indexes are more suited to primary care.

Overall, there are areas where optimal clinical care to patients with rheumatic diseases can be improved in a country-level setup where scientific evidence and international and local guidelines are governing treatment choices to individual patients. Recognition of the value of a treat-to-target strategy is widely accepted and implemented. In order to further improve the care of patients, implementation of education on the advantages and disadvantages of different tools and their optimal adaptation in different healthcare settings may contribute substantially. The continued support of national disease-based databases can provide rheumatologists with relevant data on clinical outcomes and further show the results of treatment strategies and other aspects of the structured process of managing chronic diseases. Eventually, additional involvement of patients and the integration of patient-reported outcomes in this process, as well as platforms that may offer simplicity and time-saving (eg, digital) applications, may also contribute to reaching the goal of optimal care in these patients.

### Strengths

This questionnaire was completed by 160 practicing rheumatologists from different geographical areas and settings, generally representative of the total clinicians. To our knowledge, this is one of the largest numbers of rheumatologists to have participated in such a survey in Greece. This large number of rheumatologists, as well the inclusion of doctors in a balanced ratio regarding the workplace and residence, which is representative of the total community, strengthens the results.

The questions were developed to spontaneously reflect current practice and perception rather than to investigate the application or not of specific tools in patient records. Finally, responses from this questionnaire should directly reflect real-life individual application of a treatment strategy that is facing obstacles worldwide, and therefore could potentially be the base for further studies to understand both global needs and local particularities.

### Limitations

Interindividual clinical variance may be difficult to record with prespecified questions and answers; because no open questions were available, more qualitative aspects were not recorded. Additionally, the phrasing of questions and possible answers were limited because of a limited character count per question of the platform used; therefore, the most established terms were not always used, resulting in responses that may lead to individual interpretations.

Additionally, the questions, although created along with clinicians with experience in both clinical and basic research, were created ad hoc instead of with a prespecified questionnaire, and a maximum of only one or two answers were allowed; therefore, nuances may be harder to capture. Finally, the spontaneous reporting of preferences and perceptions, without validation from actual clinical practice by patient files, could have reflected the beliefs rather the actual practice patterns of the physicians who participated.

## ABBREVIATIONS

ASDAS: Ankylosing Spondylitis Disease Activity Score
AxSpA: Axial spondyloarthritides
BASDAI: Bath Ankylosing Spondylitis Activity Disease Activity Index
CRP: C-reactive protein
DAPSA: Disease Activity index for PSoriatic Arthritis
DAS28: Disease Activity Score in 28 joints
MDA: Minimal disease activitya
PASI: Psoriasis Area and Severity Index
PsA: Psoriatic arthritis
RA: Rheumatoid arthritis
SpA: Spondyloarthritides
VAS: Visual Analogue Scale
